# Early diagnosis and intervention for autism spectrum disorder in Africa: insights from a case study

**DOI:** 10.4314/ahs.v18i1.18

**Published:** 2018-03

**Authors:** Nicola Wannenburg, Roelf van Niekerk

**Affiliations:** 1 Rhodes University, Department of psychology; 2 Nelson Mandela University, Department of Industrial and Organisational Psychology

**Keywords:** Autism spectrum disorder, case study

## Abstract

**Background:**

Globally the prevalence of autism spectrum disorder (ASD) has become more apparent. Prevalence on the African continent remains unknown. There is a call for further research into ASD in Africa as well as means to make early diagnosis and intervention possible.

**Objectives:**

The study aimed to raise awareness about ASD in Africa and encourage dialogue on possible intervention strategies for ASD in low resource settings.

**Methods:**

This was a qualitative mixed method case study. Literature regarding ASD in Africa was reviewed in order to provide context for the research and facilitate data reduction of the case study of Temple Grandin. The case study was conducted through a psychobiographical approach using Erikson's (1950/1973) theory of psychosocial development to interpret the life of Temple Grandin. The findings underwent further data reduction in order to focus on possible interventions for ASD.

**Results:**

Four primary interventions were found to be useful in facilitating development in an individual with ASD. Namely; speech therapy, creative endeavours, animals (human-animal interaction), and being mentored.

**Conclusion:**

Undiagnosed and untreated cases of ASD place undue psychosocial and economic burden on families and communities. Government support, by including ASD in health policies, is essential. Through creative adaptation of knowledge, communities may provide a valuable resource to ASD intervention strategies.

## Introduction

Awareness and concern regarding the prevalence of autism spectrum disorders (ASD) has increased worldwide. 1–3 In high resource countries, this has led to an increase in funding for ASD research and the inclusion of ASD in health policies.^1^,^4^ Despite an increase in research on ASD in Africa in the last decade,^2^,^5^ little is known about its prevalence on the continent.^5^–^7^ In part, this is owing to greater financial and research focus on communicable diseases in Africa.^6^,^8^ Non-communicable diseases, and in particular mental health,^9^,^10^ have been a low priority in legislation and public health policies. This has detracted funding from these relevant health concerns. Current research in Africa suggests that the prevalence of individuals living with ASD is greater than was previously perceived and may be increasing.^6^,^8^

The existing ASD research in Africa highlights the need for further awareness and focus on the gaps in knowledge and associated problems specific to this continent. Early diagnosis^7^,^11^,^12^ (before the age of three years) as well as interventions^13^–^15^ are imperative in trying to impede the progress of developmental delays. Early intervention increases the possibility of facilitating cognitive, physical, and psychosocial development in individuals with ASD.^3^,^7^,^16^ In African countries, diagnosis of ASD is often only made when children reach school going age, if at all.^6^,^11^,^17^ The delay in screening and diagnosis has been attributed to several factors. These include, but are not limited to, lack of knowledge of ASD among communities, care givers and professionals in the health and education sectors,^6^,^11^,^17^ as well as stigma.^8^,^18^ The financial expense^19^ of diagnostic procedures and a lack of culturally appropriate screening measures^4^,^7^,^20^ are also attributing factors.

The stigma directed at individuals displaying behaviours or symptoms related to ASD and their families may deter care givers from seeking help. There is also a belief and associated stigma that symptoms of non-communicable^21^ diseases and ASD^6^ in particular are caused by supernatural forces. This often results in care givers' primary health contact being a spiritual or traditional healer instead of a health professional that may be in a position to make a diagnosis.^17^ Late diagnosis may be related to the high incidence of non-verbal autism in Africa.^6^,^8^,^17^ This places a greater psychosocial and economic burden on families and communities of individuals with severe autism who are unable to be autonomous nor contribute to the work force.

The increased focus on ASD in Africa in the last decade suggests that health professionals' awareness and knowledge is increasing in this area. While the need for more qualified diagnosticians and easier access to ASD screening remains paramount, so is the need for financially viable interventions.^8^,^10^,^19^ More research is still required in this area.^2^,^7^ The burden of ASD remains when health professionals are able to diagnose ASD but either do not know how to manage^11^ an individual's case or are unable to assist with interventions because none are available or affordable.^1^,^8^,^17^ This may leave care givers of individuals with ASD as well as health professionals without hope. A greater focus on possible interventions that are contextually relevant may ease the burden of making a diagnosis for a disorder for which there is no cure.

Designing and implementing interventions for ASD in low resource settings may initially require adapting available information, which may have been generated in high resource settings. This information may be adapted to suit the context of the less resourced setting. Durkin et al. encourage a global flow of information and state that “autism knowledge should benefit all citizens of the world”^19^.With this in mind and the research objective of identifying possible interventions for ASD that may be viable in African countries, a long term developmental case study in the form of a psychobiography was conducted on the life of Temple Grandin. Grandin's life is contextually different in the sense of her being born in the United States of America shortly after World War II and living in a wealthy community. However, her situation with ASD shares many similar contextual difficulties to those experienced by individuals with ASD living in Africa. This is because she was born at a time when little was known about ASD and there were no established resources or interventions.^3^,^22^ It is the shared contextual challenges faced by individuals with ASD in Africa and Grandin that make focusing on interventions that aided her development potentially useful for low resource settings.

## Overview of the case study

Grandin was born on 29 August 1947. Her mother was young and inexperienced with babies as few had been born during the Second World War.^22^ Initially Grandin's parents did not notice that she was developmentally different from other babies. By the time Grandin was six months old she did not want to be held and would stiffen and pull away. A few months later it became almost impossible for anyone to hold her as she would scream, claw at the person, and struggle to free herself.^22^ Grandin was on the severe end of the autism spectrum as a child, having non-verbal autism.^22^–^24^ Despite her disruptive behaviour, withdrawn state and inability to interact or talk, Grandin's mother initially ignored her child's problems as a result of the shame the family was experiencing within their community and extended family.^22^ After receiving encouragement and advice from a friend and her 98 year old aunt, Grandin's mother sought medical help. However, little was known in society or by medical professionals about ASD at the time. This led to an initial diagnosis of infantile schizophrenia and a recommendation that Grandin's parents have her institutionalised.^22^–^24^ Grandin's mother wanted to raise her at home and tried various means to encourage Grandin to interact. This caused conflict between Grandin's parents that would later lead to them separating.^22^ Grandin's father's fury increased and he became verbally and physically abusive towards her.^22^ Grandin's mother continued to seek help from various professionals. However, they did not know what was wrong with Grandin nor how to manage her symptoms. This often resulted in them frightening Grandin in order to correct her behaviour.^22^–^25^ At the age of five, Grandin's only means of communicating were screaming, spitting and humming.^22^,^23^ She was enrolled in a small school for retarded children run by a speech therapist. At this school she began to learn to communicate, which resulted in her being allowed to attend the local country school.

When Grandin's parents enrolled her in a main stream high school she struggled both academically and socially. 22–23 She was teased for her peculiar way of walking, her mannerisms, and inability to speak normally. As a result, she got involved in fights and was expelled from school. She was then enrolled in a small farm high school with other children who had various disorders. Two of Grandin's teachers and her aunt mentored her during this time.^23^–^25^ As Grandin had begun to regress, their patience as well as creative ways of interacting with her facilitated a turning point in her development. According to Grandin animals and art enabled her to cope from an early age.^23^–^27^ Her science teacher witnessed the value of her ease around animals and fascination with inventions. He encouraged her to focus her fascination and to pursue a career in animal science.^23^,^25^ Despite the severity of Grandin's case she developed cognitively and psychosocially to the extent that as an adult she lives an autonomous and productive life as an animal scientist and autism activist.^25^–^27^ Grandin's development was facilitated by interventions. However, they were not established ASD interventions and at times required creative adaptation from members of her family and community. These interventions and their possible adaptation for ASD in African countries will structure the focus of this research.

## Methods

This research was conducted within an interpretive qualitative paradigm that employed a mixed method approach. 28 The mixed method approach is often viewed as being a combination of qualitative and quantitative methodologies. However, it may also refer to an integration of qualitative approaches.^29^,^30^ A preliminary literature review was conducted on peer-reviewed articles on ASD on the African continent to provide context and ascertain areas requiring further research.Three databases (Google Scholar, African Journals Online, and PubMed) were searched using the terms “autism” AND “autistic” AND “Africa”. Reference lists of selected articles were reviewed for further information on the topic.

### Participant and sampling method

Grandin was chosen as the subject of the psychobiographical case study by means of purposive sampling^31^ as she is an individual with ASD who developed and was able to become an internationally recognised scientist in spite of her diagnosis. She was also purposively sampled as she is an individual who had non-verbal autism as a child but later acquired the ability to speak and gave detailed accounts of her experiences. Grandin is also able to reflect upon what was useful to her development, which is rare in cases of ASD.

### Data collection

The data collection regarding Grandin's life and development was guided by the data triangulation principle.^32^,^33^ Data was therefore purposively selected from a variety of sources and carefully sort through for relevant and valid information.^32^,^33^ The collected data^22^–^27^,^34^–^43^ was also screened for its objectivity or lack thereof toward the subject of the study.

### Data processing and analysis

The data processing and analysis guidelines for this study were provided by Miles and Huberman.^44^–^46^ They^44^ propose a three step interwoven process that involves data reduction, data display, as well as conclusion drawing and verification. The conduction of the processing and analysis of data was aided by Alexander's^47^,^48^ method of asking the data questions, as well as an integration of Yin's^32^ time series analysis with Erikson's^49^ triple bookkeeping approach. Erikson's^49^–^52^ theories of psychosocial development and autism were utilized for the theoretical conceptualization and psychological analysis. The contextual information gathered during the preliminary literature review aided further data reduction and conclusion drawing on the findings of the case study that pertained to interventions for ASD that may be relevant in low resource settings.

### Ethical considerations

Informed consent was obtained from Temple Grandin for the conduction of the psychobiographical study. This consent included potential publication of journal articles. Ethical clearance was obtained from the Rhodes University Psychology Department Research Projects and Ethics Review Committee.

## Results

The main body of research findings surrounding ASD in Africa was presented in the introductory literature review to provide context to the research. Salient points are highlighted in this results section.

### Salient points/findings from the review of ASD in Africa

1. Prevalence of ASD in Africa is unknown, but is thought to be higher than previously perceived.
2. High incidence of non-verbal autism which is associated with decreased autonomy and inability to work.
3. General lack of awareness and knowledge about ASD in communities, including health professionals and educators.
4. ASD has not received necessary policy and legislation attention among governments.
5. Misconceptions surrounding the cause of ASD symptoms. Symptoms often related to supernatural forces, causing parents to seek the help of spiritual healers.
6. Stigma and shame surrounding symptoms and behaviours associated with ASD. This negatively influences the complex family dynamics of living with an individual with ASD.
7. Lack of diagnosticians and affordable and culturally appropriate screening tools.
8. Lack of resources and established interventions for ASD.
9. Insufficient number of health care professionals and educators trained to manage ASD.
10. Some medical professionals' knowledge of ASD is increasing. Generally insufficient medical and educational resources and psychosocial support for individuals with ASD as well as their care givers.

### Findings from the case study of Temple Grandin

Despite undeniable differences in geographic location and financial situation, Grandin's case shares common challenges faced by individuals living in African countries with ASD. The findings of the case study revealed five key areas regarding intervention that may aid development in an individual with ASD.They are; early and continuous intervention, speech therapy, play and creative endeavours, as well as animals and mentors. These interventions may be more adaptable and financially viable for low resource settings than established interventions and therapies for ASD.

### Early and continuous intervention

Grandin's case highlighted the role of early and continuous intervention in the development of individuals who present with severe ASD. However, it also made apparent that early intervention may not always be possible, for reasons such as parents having insufficient knowledge of autism or ignoring the extent of their child's problems because of their own shame surrounding what may be wrong with the child. Despite Grandin coming from a wealthy family, who therefore had easier access to potential interventions, her case reveals that not all interventions will work. This study did, however, reveal that learning to trust the environment and people in it are necessary for an individual with ASD to become less withdrawn and begin to interact. While consistency and reliability of people and the environment are needed by all infants in order to develop trust,^49^,^51^ these conditions may be even more necessary for individuals with ASD.

As a toddler, Grandin did not interact with her caregivers and resisted physical contact. The first active intervention her mother tried was to exchange plastic cups with Grandin when she was three years old. This required patience and continued effort on her mother's part until Grandin one day ‘noticed’ the cups and picked one up. After months, Grandin began to learn to take a cup from her mother and then give one back. Interventions which may encourage children to test the environment and begin to interact, as Grandin did when she exchanged cups with her mother, were necessary for various reasons. Firstly, it enabled Grandin to experience herself as an active agent in the world, separate from another, yet part of a community. It also facilitated the development of a mutuality of recognition relationship with another which is integral to developing hope, trust, an identity and being able to contribute to society.^49^,^50^,^53^ Interventions may need to consistently continue, and change (to suit the child's developmental stage) throughout childhood and adolescence, as was the case for Grandin.

### Speech therapy

Speech therapy was particularly useful to Grandin's psychosocial development.^49^ Even when she could not speak, learning and understanding more words enabled her to begin to understand the difference between right and wrong. This facilitated the development of more socially acceptable behaviours. At the age of five years, Grandin began to learn to speak as a result of speech therapy. This encouraged the development of her autonomy, identity, and being able to interact with and form relationships with others. Grandin's speech development took many years and even as an adult she has needed to continually work on her manner of speaking and use of words.

Grandin's speech development facilitated her development of self-reflexivity as an adolescent. During adulthood, this reflexivity aided her self-interventions and development such as learning social rules and working on her speech patterns. Grandin was also able to reflect on what made social interactions easier for her, how she could make friends and what eased her anxieties, such as being around animals. Realistically, this level of reflexivity may not be possible for all individuals with ASD. However, Grandin's adulthood makes apparent that continued interventions as well as certain consistencies of environment, people, animals, and a safe place may still be needed for the adult with ASD to function and continue to develop.

### Play and creative endeavours

Play and creativity also facilitated Grandin's development. This study revealed that there may be an association between the development of hope and resilience with play and imaginative endeavours. It is important to stress the value that play had on the development of an individual such as Grandin with non-verbal ASD who was absent and did not interact with her care givers. Grandin's play did not always look like play, but rather random repetitive action or a fixation. However, autocosmic play, such as letting sand slip through her fingers, enabled her to begin to interact with her environment and integrate experience. 52,53 Children with ASD often have difficulties with physicality. Grandin's experiences reveal that being allowed to repetitively play with objects such as newspaper, sand, or cups facilitated her learning to differentiate the boundary of her body from that of other objects and locate herself within time and space.^52^ Grandin's exchange of the cups with her mother may be viewed as a form of play. Despite repeatedly retreating to her absent state, the interplay with her mother of exchanging the cups enabled mutuality of recognition to be formed.^52^,^53^ This is of significance in an individual with autism.

Grandin was not at the same developmental level as the other children in her community. However, as a young child playing with other children who did not have ASD provided Grandin with opportunities to learn to interact and practice communicating. Grandin enjoyed playing and in order to continue playing with others she needed to remain present and recognize other individuals.

Grandin struggled at school as her speech was underdeveloped and as a result of her delayed cognitive development, she was immature for her age. Through her imaginative play, Grandin developed her creativity. She was particularly good at tasks such as sewing, drawing and woodwork, despite her difficulties in other school tasks. Although her creativity was an isolated capacity, it enabled her to demonstrate a skill at school and she managed to incorporate her ability for drawing meaningfully into her career as an adult. The benefits of her creativity to healthy personality development were in relation to (a) communicating her imaginings which is significant in individuals who struggle to speak and communicate, (b) developing a stable identity and coherent sense of self, (c) creating reflexive knowledge, and (d) increasing her selfworth and sense of purpose.

### Human-animal interaction

This study revealed that animals may assist with the negotiation and resolution of developmental tasks as well as facilitate healing in an individual with ASD. As a child, Grandin's interaction with animals aided her development of mutuality of recognition, presence, and awareness of others' needs. As Grandin's interactions with companion and farm animals increased in high school, she became more aware of her own feelings such as being able to feel attachment to another living being and learnt to be empathetic.

This was a psychological study and therefore the researchers cannot speak in scientific terms to the physiological benefits of humans interacting with animals. However, the findings do suggest that animals can help to calm the physical symptoms of an individual's anxiety and encourage physical activity and wellbeing. In terms of individuals with ASD, who struggle withphysicality and being overwhelmed by their senses, animals may facilitate the bodily integration and even enjoyment of touch or physicality.

Although Grandin transitioned from communication being impossible to always being difficult in varying degrees, from a young age she was able to communicate with animals and establish mutuality of recognition relationships. She did not always have such mutual communication and understanding with other humans. The research suggests that animals do not require the same level of verbal communication as humans do to interact. They themselves are able to interact without the use of words. The ability of animals to communicate effectively without words (or even sound) may be appealing to some individuals with autism whose senses may feel overwhelmed.

### Mentors

The analysis of Grandin's life demonstrated the significance of mentors or role models. Significant individuals may also have a negative influence on development as was evident when Grandin internalised and modeled her father's inappropriate and aggressive ways of crossing boundaries. Potential role models such as family members and teachers who were impatient and even abusive towards Grandin as a result of her difficulties increased her anxiety, decreased her self-esteem, and maintained her hyper-vigilance. Conversely, Grandin's relationships with teachers, Mr Brooks and Mr Carlock and her aunt displayed that mentors may aid the circumvention of identity diffusion^49^,^50^,^52^ and facilitate psychosocial development.

Mentors may play a significant role at various stages in an individual's development. In Grandin's case, mentors came into her life during adolescence and supported her into young adulthood. For Grandin, this was a crucial time as adolescence was particularly difficult and she had begun to regress. This research made apparent the beneficial role mentors may play in the development and support of an individual with ASD such as (a) facilitating the generational nature of the human life cycle^49^,^50^ (b) being trustworthy and empathic and thereby providing stability for a young person with developmental difficulties to negotiate and resolve previous developmental tasks, (c) being aware of an individual's difficulties as well as talents and encouraging the development of the latter, (d) providing nurturing guidance which may assist with interpersonal relationships and career choices, as well as (e) providing a model of socially appropriate and ethical behaviour.

Mentors may be beneficial to any individual. However, this case made apparent that the role they may perform in the life of an individual such as Grandin, who was often not accepted by others because of her difference, struggled with socially acceptable delineation, and had a difficult home and school life, may be of increased significance and necessity. It was also revealed that if the individual with ASD has managed to form a meaningful and trusting relationship with a mentor, continued support from the mentor into adulthood may (a) prevent regression and (b) further encourage the development of a stable identity. While not all individuals with autism may reach the level of psychosocial development necessary to become mentors themselves, Grandin's involvement as a mentor encouraged her self-awareness, empathy, self-worth, and sense of purpose. Therefore while being mentored may be essential to cognitive and psychosocial development, in turn becoming a mentor may also have psychosocial value.

## Discussion and recommendations

Four foremost areas regarding ASD in Africa were found that require further research and timely attention. These are the prevalence rate of ASD in African countries, the lack of trained diagnosticians and affordable culturally appropriate screening tools, as well as feasible interventions. Individuals with ASD living in Africa and their care givers currently face problems with diagnosis, intervention, delayed help seeking as a result of stigma and misconceptions about the cause of ASD symptoms. These problems resonate with those faced by the subject of the case study. As previously mentioned Durkin et al.^19^ pose the idea of encouraging a global flow of knowledge regarding ASD. With this in mind the discussion and recommendations that follow attempt to address problematic areas and needs regarding ASD in Africa that were highlighted in the literature review. This will be done by combining the contextual issues of ASD in African countries with information derived from the case study as well as current ASD research. While the findings of the case study cannot be generalized to others who suffer from ASD, they do provide theoretical knowledge and insights (i.e. analytical generalization) regarding interventions. The case study could also facilitate the generation of hypotheses and may be valuable to future autism research within Africa. It is hoped that the findings of this study and recommendations that follow will increase dialogue among health professionals, educators as well as caregivers surrounding possible interventions for ASD in low resource settings.

The findings of this study support the available research that early diagnosis^7^,^11^,^12^ and intervention^13^–^15^ are imperative to facilitating development in individuals with ASD. This highlights the need for health and governmental policies in African countries that take ASD into consideration to improve the training of health workers and increase screening and intervention resources. Increased awareness in communities about ASD may decrease stigma and encourage care givers to seek help for children displaying symptoms of ASD. As care givers often seek the help of traditional or spiritual healers,^6^,^17^,^21^ it is suggested that such healers who would be willing to learn more about ASD be incorporated into health management teams in communities. Durkin et al.^19^ suggested open access of diagnostic tools to facilitate screening in low resource and economic settings. While this would be beneficial, it may take years before this becomes a reality. At present the 23-question screener (23Q) developed by Kakooza-Mwesigeet al.^20^ in Uganda shows promise as a financially viable screening measure for ASD that considers cultural and contextual factors. Further research regarding the applicability of the 23Q for diagnosing ASD in other African countries may increase awareness and refinement of this screening measure.

The research findings support related research that speech therapy^13^,^17^,^54^ and learning communication skills are imperative for non-verbal ASD and may be beneficial in the cases of verbal ASD. Speech therapy is expensive and there are few trained speech therapists in Africa.^6^ For individuals with ASD learning to communicate in some way may facilitate autonomy and ability to contribute to the home environment, society, and the economy. Therefore this particular intervention (speech therapy) for ASD should receive greater consideration from governments and their funding policies. Teaching families of children with ASD as well as educators and peers communication strategies has shown to be useful.^54^–^57^ Workshops by existing or visiting speech and language professionals on strategies to communicate with individuals with ASD may be considered a viable option in some settings.

In line with the research findings, development in general, and more specifically communication and behavioural skills have shown to improve in children with ASD when they are mentored either by adults or peers.^55^–^57^ Art programs^58^–^61^ are also being used to facilitate a means of communication in children with ASD and the development of reflexivity. The research into animal assisted activities for children with ASD is still limited.^62^ However, current research^63^ and the findings of the case study suggest that interacting with animals may be emotionally as well as cognitively beneficial to children with ASD. While children with ASD can be encouraged to create things and interact with animals at home, community involvement may further facilitate the possibility of such interventions. Communities are filled with resources within themselves. Willing members of communities could set up mentoring programs specifically for children with ASD, while art teachers or local artists could run art programs. Rural settings are often less resourced than their urban counterparts. Nevertheless, rural settings may provide more opportunities for middle to high functioning individuals with ASD to work with animals, which may be easier for them than working with other people. It is recommended that people willing to take part in such community programs first increase their knowledge surrounding ASD.

## Conclusion

A review of the current state of ASD in Africa was provided. This highlighted the need for early diagnosis and financially feasible interventions on the continent. The findings from the case study of Temple Grandin revealed possible intervention strategies for an individual with ASD. Further recommendations as to how these interventions may be adapted in low resource settings were provided. It is hoped that this study will encourage further research and dialogue on ASD in Africa and the resources available within communities.
